# Human Immunodeficiency Virus and Allergic Bronchopulmonary Aspergillosis: Case Report and Review of Literature

**DOI:** 10.1093/ofid/ofw116

**Published:** 2016-06-20

**Authors:** Panagis Galiatsatos, Michael T. Melia, Leann L. Silhan

**Affiliations:** 1 Critical Care Medicine, National Institutes of Health, Bethesda; 2 Divisions of Infectious Diseases; 3 Pulmonary and Critical Care Medicine, Johns Hopkins Medicine, Baltimore, Maryland

**Keywords:** allergic bronchopulmonary aspergillosis, bronchiectasis, HIV infection

## Abstract

Allergic bronchopulmonary aspergillosis (ABPA) results from a hypersensitivity response to airways colonization with *Aspergillus fumigatus*, and it occurs most often in individuals with asthma or cystic fibrosis. Allergic bronchopulmonary aspergillosis is an indolent, but potentially progressive, disease in patients. In patients infected with human immunodeficiency virus (HIV), ABPA is rare, and its description in the literature is limited to case reports. We describe the occurrence of ABPA in a 37-year-old woman with well controlled HIV infection. This represents the first documented case of ABPA in an HIV-infected patient whose only pulmonary comorbidity included the ramifications of prior acute respiratory distress syndrome due to *Pneumocystis jirovecii* pneumonia. We also review prior case reports of ABPA in HIV-infected patients and consider risk factors for its development.

*Aspergillus* species are common fungi found throughout the world. Their spores thrive in humid, organic material. In humans, the pathogenicity of *Aspergillus* species is variable and depends on host characteristics [1]. For instance, invasive aspergillosis is commonly found in immunocompromised patients, whereas chronic necrotizing aspergillosis is often found in patients with chronic obstructive pulmonary disease, prior tuberculosis, or current corticosteroid therapy [1]. Another entity triggered by *Aspergillus*, allergic bronchopulmonary aspergillosis (ABPA), occurs almost exclusively in patients with asthma or cystic fibrosis; atopy is another risk factor [1]. In ABPA, the presence of *Aspergillus fumigatus* antigens in the airways leads to a complex allergic response involving both innate and adaptive portions of the immune system [2].

The impact of human immunodeficiency virus (HIV) on the immune system has been well described [3]. With the initiation of antiretroviral therapy (ART), there is marked improvement in the cellular immunity of patients with HIV infection [4]. However, even with a restored immune system, the discussion of allergies in HIV-infected persons is complex. For example, atopic diatheses have been reported in HIV patients but mainly in children [5] and in patients with acquired immune deficiency syndrome [6]. The spectrum of atopy in HIV patients ranges from dermatitis to asthma [7]. However, ABPA is a rare phenomenon and, to date, it has only been case reported in HIV-infected patients. We present a case of a patient with HIV and a new diagnosis of ABPA, whose only prior pulmonary comorbidity was a severe case of acute respiratory distress syndrome (ARDS). Furthermore, we review prior cases and assess potential risk factors for the development of ABPA in HIV-infected patients.

## CASE REPORT

A 37-year-old HIV-infected woman presented to pulmonary clinic in early 2016 for evaluation of years of persistent cough and dyspnea. Her relevant medical history included a prolonged hospitalization in 2010. At that time, she was not on ART and her absolute CD4 lymphocyte count was 6/mm^3^. She was hospitalized for evaluation of dyspnea and chest pain; she was found to have bilateral pneumothoraces. She was intubated for respiratory failure and had chest tubes placed. She was found to have *Pneumocystis jirovecii* pneumonia. She required mechanical ventilation for a total of 3 months.

During her hospitalization, ART was initiated and an undetectable viral load was achieved. Although she subsequently was found to have viremia owing first to resistance mutations and later to medication nonadherence, she has maintained an undetectable HIV viral load since 2013. Her most recent CD4 lymphocyte count is 588/mm^3^. Her current ART program includes etravirine, dolutegravir, and ritonavir-boosted darunavir.

Six months after her prolonged hospitalization in 2010, she had a pulmonary function test that showed no obstruction and significant restriction (total lung capacity was 48.5% of predicted). She was using inhaled beclomethasone twice daily and an albuterol inhaler as needed at the time of testing. Upon her evaluation in the pulmonary clinic in 2016, she complained of a frequent nonproductive cough that had been persistent since her illness in 2010, and although she functionally recovered after her critical illness, she continued to have significant dyspnea on exertion. She described shortness of breath walking 1 city block or climbing 1 flight of stairs. She had occasional wheezing and reported triggers included strong odors, seasonal changes, and her current living space. After her critical illness in 2010, she moved into a rental property, which had to be renovated twice due to mold, and then in 2014 she moved to another property, in which water damage occurred from a leaking air conditioner. The carpet was taken up and dried but ultimately remained in the home. At that time, she began to feel her pulmonary symptoms worsen in regards to coughing frequency and endurance limitations; these symptoms progressed through the time of her initial pulmonary clinic appointment. She denied orthopnea or lower extremity edema. She was a life-long nonsmoker and did not use illicit drugs.

On examination, she was in no acute distress, and she was obese (body mass index of 35.4 kg/m^2^) with oxygen saturation 100% on pulse oximetry at room air with no desaturation on ambulation. On percussion, she had small lung volumes, inspiratory rhonchi over the left upper lung field, but otherwise was clear to auscultation without wheezing. There was bilateral axillary scarring from prior chest tube insertions, with keloid formation. Her cardiac and abdominal exams were unremarkable. She had no lower extremity edema or clubbing.

**Figure 1.**
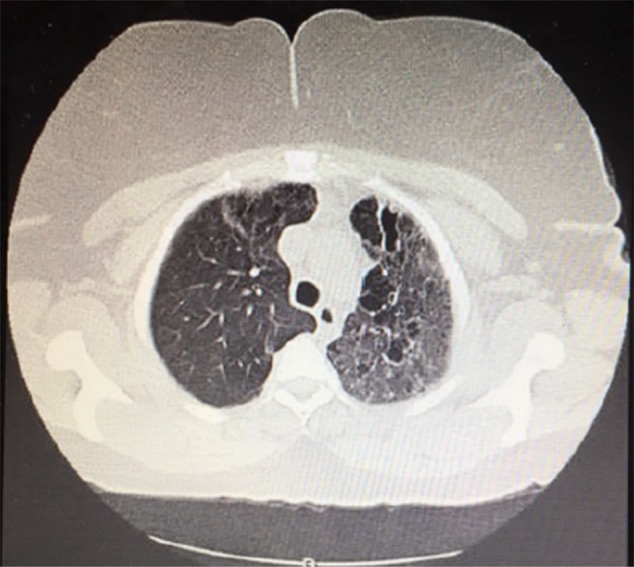
Computed tomography of the chest from our patient, revealing cystic bronchiectasis and ground-glass opacities in the left lung.

Computed tomography of the chest without contrast (Figure 1) revealed fibrotic changes predominantly in the upper lobes (left greater than right) and upper lobe ground-glass opacities. There was varicoid and cystic bronchiectasis associated with the fibrosis in the left upper lobe, right upper lobe, right middle lobe, and lingula. Calcified pleural plaques were present along bilateral hemidiaphragms. There were calcified left hilar lymph nodes and a normal cardiac examination.

Updated pulmonary function tests obtained in 2016 showed significantly improved restriction (total lung capacity of 64.6% predicted), although concomitant obstruction was now seen (forced expiratory volume at 1 second [FEV_1_] was 58.5% predicted). Complete blood count revealed mild eosinophilia of 320/mm^3^. We were able to review laboratory testing from 2010, which included an absolute eosinophil count of 640/mm^3^, before therapy with inhaled corticosteroid.

Out of concern for a diagnosis of ABPA, total serum immunoglobulin (Ig)E and *Aspergillus*-specific IgE were sent. Both were found to be markedly elevated (Table 1), supporting a diagnosis of ABPA. In addition, given her identifiable triggers that correlated with dyspneic episodes, we ran an allergy (Rast Northeast) panel, which reported elevated IgE levels to allergens that included *A fumigatus*, cat, dog, and environmental stimuli (eg, birch, oak). Given the patient's clinical history, positive mold exposure, radiographic findings, and laboratory data, a diagnosis of ABPA was suspected. The patient was started on prednisone at 0.2 mg/kg per day, which is lower than recommended for initiation for ABPA, but given the patient's HIV infection and potential drug-drug interactions, this dose was decided on in a multidisciplinary discussion. Itraconazole 200 mg daily was also started and was then adjusted based on levels.

**Table 1.** Patient Characteristics From Case Reports at Time of Diagnosis of Allergic Bronchopulmonary Aspergillosis^a^

Age	Sex	CD4 Count (Cells/mm^3^)	Antiretrovirals	Prior OI	CM	AIgE Level (kU/L)	IgE Level (kU/L)	Treatment
33^b^	Man	300	Lamivudine Indinavir Zidovudine	*Aspergillus* pneumonia	Tobacco Asthma Chronic sinusitis	3798	6822.6	Prednisone
35^c^	Woman	597	Zidovudine Lamivudine Nevirapine	None	Asthma Seasonal rhinitis	100	29 600	Itraconazole
37^d^	Woman	588	Dolutegravir Ritonavir Darunavir Etravirine	PCP	Asthma	6.66	2020	Itraconazole Prednisone

Abbreviations: AIgE, *Aspergillus* immunoglobulin E; CM, comorbidities; OI, opportunistic infection; PCP, *Pneumocystis jirovecii* pneumonia.

^a^ Note: Normal levels for *Aspergillus* IgE are <0.34 kU/L, and normal levels for IgE are ≤114 kU/L.

^b^ Patient from Reference [13].

^c^ Patient from Reference [12].

^d^ Our patient.

The patient returned after 4 months on the aforementioned regimen with significantly improved pulmonary symptoms: no coughing or wheezing, improvement in her endurance, and less usage of her as-needed inhalers. She had a new set of pulmonary function testing that revealed improvement in her FEV_1_ (60.2%), total lung capacity (69.6%), and diffusion capacity (65%; increased from 56%). Furthermore, her IgE levels had decreased from 2020 to 903 kU/L and her *A fumigatus*-specific IgE levels went from 6.6 to 3.01 kU/L.

## DISCUSSION

The pathophysiology behind ABPA is complex, with local inflammation resulting from ineffective spore removal, which in turn leads to increased mucus production, airway hyperreactivity, and bronchiectasis [8]. *Aspergillus* spores that are allowed to germinate and proliferate in the airways ultimately result in a hypersensitivity response that is thought to be due to a predominance of allergic T-cell lymphocytes (Th2) over nonallergic T-cell lymphocytes (Th1) [9]. There is also thought to be a genetic predisposition to ABPA in atopic patients, whereby patients with ABPA also have higher rates of other atopic conditions, such as allergic rhinitis, atopic dermatitis, and food sensitivities [1]. Therefore, the high prevalence of ABPA in diseases such as asthma and cystic fibrosis is logical given that these 2 conditions are strongly associated with atopy. Other diseases in which ABPA has been reported include hyper-IgE syndrome and chronic granulomatous disease [10]. However, the occurrence of ABPA in HIV-infected persons seems to be rare.

There are 2 prior published case reports of HIV-infected patients with ABPA [11, 12]. Details about these patients are summarized in Table 1. The common findings among these patients is that they were on ART, had CD4 counts above 200 cells/mm^3^, and had undetectable HIV viral loads. Like our patient, they both had prior atopic disease: asthma, allergies, and rhinitis. However, our patient differs in that her diagnosis of asthma was made after her HIV diagnosis and after an infectious pneumonia that resulted in prolonged mechanical ventilation. Furthermore, our patient was treated with dual antifungal and corticosteroid therapy, whereas the patients from the other 2 case reports were treated with only steroids or only antifungals (Table 1).

Although ABPA and HIV are rare as comorbidities, atopy and HIV seem to be more common [13, 14]. This is thought to occur due to a Th2 predominant response in HIV patients versus Th1 [15]. Our patient was discovered to have allergies through a detailed self-history report of “triggers” that caused dyspnea and by laboratory testing. She had no family history of atopy, although she did have a mild eosinophilia dating back to 2010 (range, 200–640, with normal 120–300). Finally, it is unclear how her prior infection with *P jirovecii* resulting in pneumonia and ARDS is related to her current pulmonary manifestations, both radiographically and symptomatically, because there are no reports that specifically highlight these outcomes found in our patient.

## CONCLUSIONS

In conclusion, our patient with well controlled HIV infection was diagnosed with ABPA. This highlights that ABPA can develop postlung injury that results in bronchiectasis, and, although rare with concomitant HIV infection, vigilance to diagnose ABPA is necessary in an asthmatic patient with bronchiectasis. This represents the third publication to date of ABPA and HIV occurring in the same patient, and it offers more insight into the development of this disease in a patient without a long-standing history of atopy or pulmonary disease. Furthermore, this is the first case of ABPA in an HIV patient treated with dual therapy: a corticosteroid and an antifungal agent. This case serves as an important reminder to consider diagnoses associated with a hyperactive immune response in patients with well controlled HIV infection.

## References

[1] Soubani AO, Chandrasekar PH The clinical spectrum of pulmonary aspergillosis. Chest 2002; 121:1988–99. 12065367 10.1378/chest.121.6.1988

[2] Tillie-Leblond I, Tonnel AB Allergic bronchopulmonary aspergillosis. Allergy 2005; 60:1004–13. 15969680 10.1111/j.1398-9995.2005.00887.x

[3] Streeck H, Nixon DF T cell immunity in acute HIV-1 infection. J Infec Dis 2010; 202 (Suppl 2):S302–8. 20846037 10.1086/655652 PMC2954287

[4] Sempowski GD, Haynes BF Immune reconstitution in patients with HIV infection. Annu Rev Med 2002; 53:269–84. 11818474 10.1146/annurev.med.53.082901.104032

[5] Da Silva L, Kweku Sagoe Amoah S, Da Silva J Relationship between atopy, allergic diseases and total serum IgE levels among HIV-infected children. Eur Ann Allergy Clin Immunol 2013; 45:155–9. 24129042

[6] Lange CG, Gripshover BM, Valdez H, Lederman MM [Manifestation of hyper-IgE syndrome in advanced HIV-1 infection]. Med Klin (Munich) 2002; 97:34–9. 11831060 10.1007/s00063-002-1122-3

[7] Marshall GD Jr AIDS, HIV-positive patients, and allergies. Allergy Asthma Proc 1999; 20:301–4. 10566099 10.2500/108854199778251979

[8] Denning DW, O'Driscoll BR, Hogaboam CM et al The link between fungi and severe asthma: a summary of evidence. Eur Respir J 2006; 27:615–26. 16507864 10.1183/09031936.06.00074705

[9] Knutsen AP, Chauhan B, Slavin RG Cell-mediated immunity in allergic bronchopulmonary aspergillosis. Immunol Allergy Clin North Am 1998; 18:575–99. 10.1159/000234365 2886438

[10] Riscili BP, Wood KL Noninvasive pulmonary aspergillus infections. Clin Chest Med 2009; 30:315–35. 19375638 10.1016/j.ccm.2009.02.008

[11] Jain M Allergic bronchopulmonary aspergillosis in an HIV-infected individual. Allergy Asthma Proc 2000; 21:351–4. 11191100 10.2500/108854100778249051

[12] Muthu V, Agarwal R A report of a successfully treated case of ABPA in an HIV-infected individual. BMJ Case Rep 2014; pii:bcr2014206236. 10.1136/bcr-2014-206236 PMC4225263 25385562

[13] Rudikoff D The relationship between HIV infection and atopic dermatitis. Curr Allergy Asthma Rep 2002; 2:275–81. 12044260 10.1007/s11882-002-0050-x

[14] Masekela R, Moodley T, Mahlaba N et al Atopy in HIV-infected children in Pretoria. S Afr Med 2009; 99:822–5. 20218485

[15] Maggi E, Mazzetti M, Ravina A et al Ability of HIV to promote a Th1 to Th0 shift and to replicate preferentially in Th2 and Th0 cells. Science 1994; 265:244–8. 8023142 10.1126/science.8023142

